# Miscarriage Rate Is High With Frozen-Thawed Blastocysts Arising From Poor-Quality Cleavage Stage Embryos

**DOI:** 10.3389/fendo.2020.561085

**Published:** 2020-09-16

**Authors:** Lan Xia, Shen Zhao, Huiui Xu, Xian Wu, Aijun Zhang, Zhihong Niu

**Affiliations:** Reproductive Medical Center of Ruijin Hospital, School of Medicine, Shanghai Jiao Tong University, Shanghai, China

**Keywords:** blastocysts, embryo, miscarriage rate, perinatal outcomes, clinical pregnancy rate

## Abstract

Embryos with low morphological scores can still develop to the blastocyst stage and result in good clinical outcomes. However, no studies have reported the possible effects of transferring cryopreserved blastocysts developed from poor-quality cleavage stage embryos on pregnancy and perinatal outcomes. In this retrospective study, the clinical value of transferring blastocysts derived from day 3 poor-quality cleavage stage embryos during *in vitro* fertilization and embryo transfer procedures was evaluated. According to the quality of embryos on day 3 from which the transferred blastocyst originated, patients were divided into three groups: poor-quality (111 cycles, group A), good-quality (235 cycles, group B), and top-quality (119 cycles, group C). Group A experienced the highest miscarriage rate (30.2%) which was increased when compared to group C (12.5%) (*P* = 0.03). The clinical pregnancy rates and live birth rates were not significantly different among the three groups. However, good blastocyst originating from top day 3 embryos resulted in higher live birth rate. Of the 218 live births, no differences in obstetric and perinatal outcomes were noted among the three groups. The results showed that extended culture of poor-quality cleavage stage embryos could resulted in favorable clinical pregnancy rates but at a higher incidence of miscarriages. Meanwhile, the risk of adverse perinatal outcomes was not increased.

## Introduction

The success rate of *in vitro* fertilization (IVF) is influenced by many factors such as age, infertility diagnosis, and embryo quality. Embryo quality, based on morphological parameters, is a major predictor of the success of IVF-embryo transfer (ET) during assisted reproductive technology (ART) treatment. The association between the quality of cleavage stage embryos and the implantation rate (IR) and pregnancy outcomes has been well-established (1, 2). Poor quality embryos are often discarded due to their low developmental potential. Rejecting poor-quality embryos (PQE) in the field of ART is a conflicting clinical strategy due to the low survival rate and IR. Many clinics selected good-quality embryos for transferring and discarded PQE. In addition, the cost of ART is high and patients want to spend less money and time on it. Culturing surplus PQE could avoid repeated IVF cycles, which also reduces the cost of IVF treatment and save patients' time (3).

Recently, the embryo culture systems have improved the efficiency and reliability of obtaining blastocysts *in vitro*, and PQE have been reconsidered for further developmental potential. Many findings suggest that embryos with low morphological scores can still grow into the blastocyst stage and have a good clinical outcome (3–6). However, the transfer of a good-quality embryos might improve obstetric and perinatal outcomes (5, 6). To the best of our knowledge, few studies have evaluated the possible effects of transferring cryopreserved blastocysts arising from poor-quality cleavage stage embryos on pregnancy and perinatal outcomes. Thus, this study aimed to evaluate the clinical value of transferring blastocysts arising from day 3 poor-quality cleavage stage embryos during the IVF-ET procedure.

## Materials and Methods

### Participants

This study retrospectively analyzed the pregnancy and perinatal outcomes of patients who received frozen-thawed blastocysts. Data were collected between August 2017 and January 2019 at the reproductive center of the Ruijin Hospital affiliated with the Medical school of Shanghai Jiaotong University. The inclusion criteria were as follows: (1) couples aged ≤40 years at the time of oocyte retrieval, (2) ovarian stimulation cycle was completed with a freeze-all protocol rather than a fresh ET, and (3) transfer of blastocysts instead of day 3 cleavage stage embryos in the frozen embryo transfer (FET) cycle.

The exclusion criteria included a history of pregnancy loss, abnormal karyotype, or history of pathology affecting the endometrial cavity and/or receptivity. The patients were grouped retrospectively according to the transferred blastocyst and the quality of day 3 embryos: group A, the transferred blastocysts developed from PQE on day 3; group B, the transferred blastocysts developed from good-quality embryos on day 3; and group C, the transferred blastocysts developed from top-quality embryos on day 3.

The treatment was conducted after informed consent was obtained from the patients and was carried out according to the guidelines of the Ministry of Public Health of China. It was approved by the reproductive ethics committee at the Medical school of Shanghai Jiaotong University.

### Ovarian Stimulation and Oocyte Retrieval

Patients selected for IVF were monitored and managed according to the standardized clinical protocols as previously reported (7). Briefly, ovarian stimulation was performed with human menopausal gonadotropin and recombinant follicle-stimulating hormone (FSH). The doses of gonadotropins were determined on an individual basis according to the woman's age, day 3 serum FSH value, and antral follicle count. Pituitary inhibition was obtained by gonadotropin-releasing hormone (GnRH) agonist or GnRH antagonist. When three or more dominant follicles reached 17–18 mm, 5000 IU of human chorionic gonadotropin (hCG) was administered. Oocyte retrieval was performed transvaginally under ultrasound guidance at 34–36 h after hCG injection.

### IVF and Embryo Culture

Based on the semen quality on the day of oocyte retrieval, the oocytes were inseminated 3–6 h after oocyte retrieval with either intracytoplasmic sperm injection (ICSI) or conventional insemination. Fertilization was indicated by the appearance of two distinct pronuclei and two polar bodies 16–18 h after insemination. The zygotes were cultured individually in the cleavage medium (G-1 PLUS, Vitrolife, G Gothenburg, Sweden) overlaid with oil. Blastocysts were cultured in the cleavage medium for the first 72 h and subsequently in the blastocyst medium (G-2, Vitrolife, Gothenburg, Sweden) overlaid with oil in a humidified 6% CO_2_ 5% O_2_ at 37°C until days 5–6.

### Embryo Quality

Embryo evaluation was carried out on day 2 (44–46 h) and day 3 (68–70 h) using the 200X magnification of an Olympus microscope. Embryos with multinucleation were excluded from this study and were defined as the presence of more than a single interphase nucleus in a blastomeric. Embryos were scored on day 3 based on following three criteria: (A) Blastomere number (BL): 1 = 4 BL; 2 = 5 BL; 3 = 6–7 BL, and 4 = 8–10 BL; (B) Fragmentation (FR) scores: 4 = <5% FR, 3 = 5–10% FR, 2 = 11–25% FR, 1 = 26–50% FR, 0 = > 50% FR; and (C) Symmetry (SY) scores, score 1, perfect symmetry, and score 0, severe asymmetry. The total score for an embryo was the summation of the three parameters (BL, FR, and SY) and the group criteria was: PQE, score <5; good-quality embryo, score 5–7 with three to five cells at day 2; and top-quality embryo, score 8–10 with four cells at day 2. In our center, embryos of the top four qualities on the third day were frozen, while the remaining embryos underwent extended culture which were frozen at blastocyst stage if they developed to blastocyst.

On the days 5–7 after oocyte retrieval, the blastocysts were scored according to Gardner (8): stage 1, early blastocyst with a blastocoel less than one-half volume of that of the embryo; stage 2, with a blastocoel one-half volume of that of the embryo or more; stage 3, full blastocyst with a blastocoel completely filling the embryo; stage 4, expanded blastocyst with a blastocoel volume larger than that of the blastocyst and a thinning zona; stage 5, hatching blastocyst with a trophectoderm starting to herniate through the zone and stage 6, hatched blastocyst. The inner cell mass (ICM) grading was as follows: (A) tightly packed many cells; (B) loosely grouped, several cells; and (C) very few cells. The trophectoderm (TE) grading was as follows: (A) many cells forming a tightly knit epithelium, (B) few cells, and (C) very few cells forming a loose epithelium. Good quality blastocysts were defined as 3-6AA, 3-6AB, 3-6BA, and 3-6BB. Fair quality blastocysts were defined as 3-6BC and 3-6CB. For statistical convenience and the effect of embryo quality on clinical outcomes, we quantified the transferred expanded blastocyst score as follows: AA was scored 10, AB/BA was scored 9, BB was scored 8, AC/CA was scored 7, and BC/CB was scored 6. All evaluations of embryos were double checked by two embryologists.

### Vitrification Cooling and Warming Protocol

Vitrification and warming protocols were conducted following traditional methods according to the instructions of the Vit Kit (Kitazato Biopharma, Japan). Moreover, one to two embryos were progressively incubated from 8 min (cleavage stage embryos) to 10 min (blastocysts) in 20-μl equilibration solution (7.5% v/v of each dimethyl sulfoxide [DMSO] and ethylene glycol), followed by two incubation cycles for 5 s and one incubation cycle for 10 s in 20 μl vitrification solution (VS; 15% v/v of each DMSO and ethylene glycol, 0.5 M sucrose). The embryos were submerged in VS for 60 s. The smallest possible volume of the vs. containing the embryo(s) was loaded into the Cryotop and then plunged horizontally into liquid nitrogen (LN2).

During warming, the Cryotop was removed from liquid nitrogen, and the embryo(s) was immersed in thawing solution (1.0 M sucrose) for 1 min, then transferred into 20 μl of dilution media (0.5 M sucrose) for 3 min, followed by two incubation cycles at 3 min and washed at room temperature. During the last incubation step, embryos were brought progressively back to 37°C and cultured for 2 h in the cleavage medium and the blastocyst medium. Survival of embryos was monitored immediately after the warming procedure and before transfer.

### Endometrial Preparation

In our study, we prepared the endometrium using hormone replacement treatment (HRT). Estradiol valerate (4 mg; Progynon, Bayer) was administered to patients daily for 10 days starting on cycle day 3. Then, the E2 valerate dose was adjusted according to the endometrial thickness. The date to perform the vitrified/warmed blastocyst transfer was determined according to endometrial thickness and the results of a serum hormone test. Progesterone supplementation started 5 days prior to blastocyst transfer and continued until a pregnancy test was performed. If the test was positive, progesterone supplementation continued for an additional 5 weeks.

### Pregnancy Outcomes and Perinatal Outcomes

Serum β-hCG levels were measured 9 days after blastocyst transfer. The IR, clinical pregnancy rate (CPR), live birth rate (LBR) and miscarriage rate were recorded. Clinical pregnancy was defined as the presence of a gestational sac observed in an ultrasound scan at around 7 weeks of amenorrhea. Miscarriage was defined as the loss of a clinical pregnancy before 20 completed weeks of gestational age. All pregnancies were tracked until delivery. Adverse neonatal outcomes included preterm delivery (PTD before 37 weeks), low birth weight (LBW, birth weight <2,500 g), congenital anomalies, and perinatal mortality.

### Statistical Analysis

All analyses were performed using SPSS 22.0 software (IBM Corp., Armonk, NY, USA). Categorical variables are expressed as *n* (%), and chi-square test was used to compare differences between groups. Continuous variables are expressed as mean ± standard deviation or median (interquartile range). Wilcoxon rank sum tests or *t*-tests were performed for continuous variables where appropriate. A *P*-value of < 0.05 was considered a statistically significant difference.

## Results

### Patient Characteristics in Fresh Cycles

A total of 465 cycles were collected and compared. The demographics of patients' experiencing fresh cycles are shown in Table 1. The maternal and paternal age ranged from 26 to 40 years, and from 27 to 45 years, respectively. The duration of infertility ranged from 1 to 10 years. The overall characteristics of the patients, including type of infertility, basal FSH, days of ovarian stimulation, and assisted reproduction techniques were similar. However, the number of retrieved oocytes, fertilization rate, and cleavage rate were comparable among the three groups (Table 1).

**Table 1:** Patients' demographics in fresh cycles.

	**Group A (*n* = 111)**	**Group B (*n* = 235)**	**Group C (*n* = 119)**	***P-*value**
Maternal age (year)	33.3 ± 4.4	32.9 ± 4.7	33.9 ± 4.3	0.21
25~30 (*n*, %)	31 (27.9)	67 (28.5)	32 (26.8)	0.95
31~35 (*n*, %)	49 (44.1)	105 (44.7)	47 (39.5)	0.63
36~40 (*n*, %)	31 (27.9)	63 (26.8)	40 (33.6)	0.40
Paternal age (year)	34.7 ± 5.0	34.9 ± 5.5	35.6 ± 5.7	0.39
BMI (kg/m^2^)	22.6 ± 1.6	22.3 ± 1.7	22.4 ± 1.5	
Duration of subfertility (years)	3.4 ± 2.3	3.7 ± 2.9	3.8 ± 3.1	0.58
**Cause of infertility**				
Tube (*n*, %)	75 (67.6)	152 (64.7)	73 (61.3)	0.42
Endometriosis (*n*, %)	8 (7.2)	24 (10.2)	9 (7.6)	0.37
Oligozoospermia (*n*, %)	28 (25.2)	59 (25.1)	37 (31.1)	0.55
Primary subfertility (%)	62 (55.9)	136 (57.9)	59 (49.6)	0.33
ICSI (%)	39 (35.1)	73 (31.1)	44 (37.0)	0.50
Basal FSH (IU/L)	6.6 ± 1.5	6.5 ± 2.1	6.8 ± 1.6	0.70
**Ovarian stimulation protocol**				
GnRH agonist (%)	35 (24.7)	62 (26.4)	38 (31.9)	0.62
GnRH antagonist (%)	76 (75.3)	173 (73.6)	81 (68.1)	0.44
Starting dose of gonadotropins (IU)	225 (150, 300)	225 (150, 300)	225 (150, 300)	0.37
Total dose of gonadotropins (IU)	2,475 (1,500, 3,600)	2,500 (1,350, 3,600)	2,425 (1,425, 3,450)	0.29
Days of ovarian stimulation	10.5 ± 2.2	10.3 ± 1.6	10.0 ± 1.5	0.13
No. of oocytes retrieved	11.3 ± 5.4	12.3 ± 5.2	11.9 ± 5.4	0.26
Fertilization rate	1001/1253	2360/2889	1180/1417	0.08
	(78.6%)	(81.7%)	(83.3%)	
Cleavage rate	996/1001 (99.5%)	2347/2360 (99.5%)	1177/1180 (99.8%)	0.46

### Blastocyst Quality of the Three Groups

To determine the origin of the embryonic differences among the three groups, we compared the viable rate and good-quality blastulation rates (Table 2). The overall blastulation rate eligible for vitrification was 38.28%, and the good-quality blastulation rate was 6.88%. Both the viable and good-quality blastulation rates were lower in group A than in other groups.

**Table 2:** Blastulation rates of the three groups.

	**Group A (*n* = 111)**	**Group B (*n* = 235)**	**Group C (*n* = 119)**
Cultured embryos	750	1602	903
Viable blastulation rate^a^	169/750 (22.5%)	598/1602 (37.3%)	379/903 (42.0%)
Good-quality blastulation rate^b^	36/750 (4.8%)	112/1602 (7.0%)	76/903 (8.4%)

a *The difference is significance among the three groups (group A vs. group B, p < 0.01. group A vs. group C, p < 0.01, group B vs. group C, p = 0.02)*.

b *The difference is significant between group A and group C (p = 0.01)*.

### Characteristics of Patients Undergoing FET Cycles

The demographics of patients' undergoing the FET cycles are shown in Table 3. The survival rate of all embryos was 96.59% (594/615). The overall single blastocyst transfer rate was 72.26% (336/465), and the proportion was equivalent among the three groups (68.5, 74, and 72.3%). There were no differences in the mean number and the quality of the transferred blastocysts quantified by the score of the extended embryos, including the degree of expansion (stages 3–6), quality of the ICM and the TE (A/B) among the three groups, respectively. In the double embryos transferred cases, both blastocysts were originated from the same class of cleavage stage embryos.

**Table 3:** Parameters in the frozen embryo transfer cycles of the three groups.

	**Group A (*n* = 111)**	**Group B (*n* = 235)**	**Group C (*n* = 119)**	***P*-value**
Endometrium thickness on progesterone initial day (mm)	10.0 ± 1.0	10.0 ± 1.1	10.1 ± 1.1	0.97
E_2_ level on progesterone initial day (pg/ml)	217.2 ± 23.1	219.0 ± 20.9	222.6 ± 26.4	0.22
P level on progesterone initial day (ng/ml)	0.61 ± 0.06	0.62 ± 0.06	0.62 ± 0.06	0.63
Duration of cyto-storage (month)	8(2,12)	7(1–14)	8(2–14)	
Post-thaw embryo survival rate	148/152 (97.4%)	296/306 (96.7%)	152/157 (96.8%)	0.92
No. of transferred embryos (*n*)	1.3 ± 0.5	1.3 ± 0.6	1.3 ± 0.5	0.55
Single blastocyst transfers, % (*n*)	76/111	174/235	86/119	0.56
	(68.5%)	(74.0%)	(72.3%)	
Transferred blastocyst score	6.7 ± 0.9	6.8 ± 1.0	6.9 ± 1.0	0.13
Origin embryo score (mean)	3.9 ± 0.1	6.1 ± 0.8	8.6 ± 0.3	<0.01

*E_2_, serum estradiol; P, serum progesterone*.

### Pregnancy Outcomes

**Figure 1:**
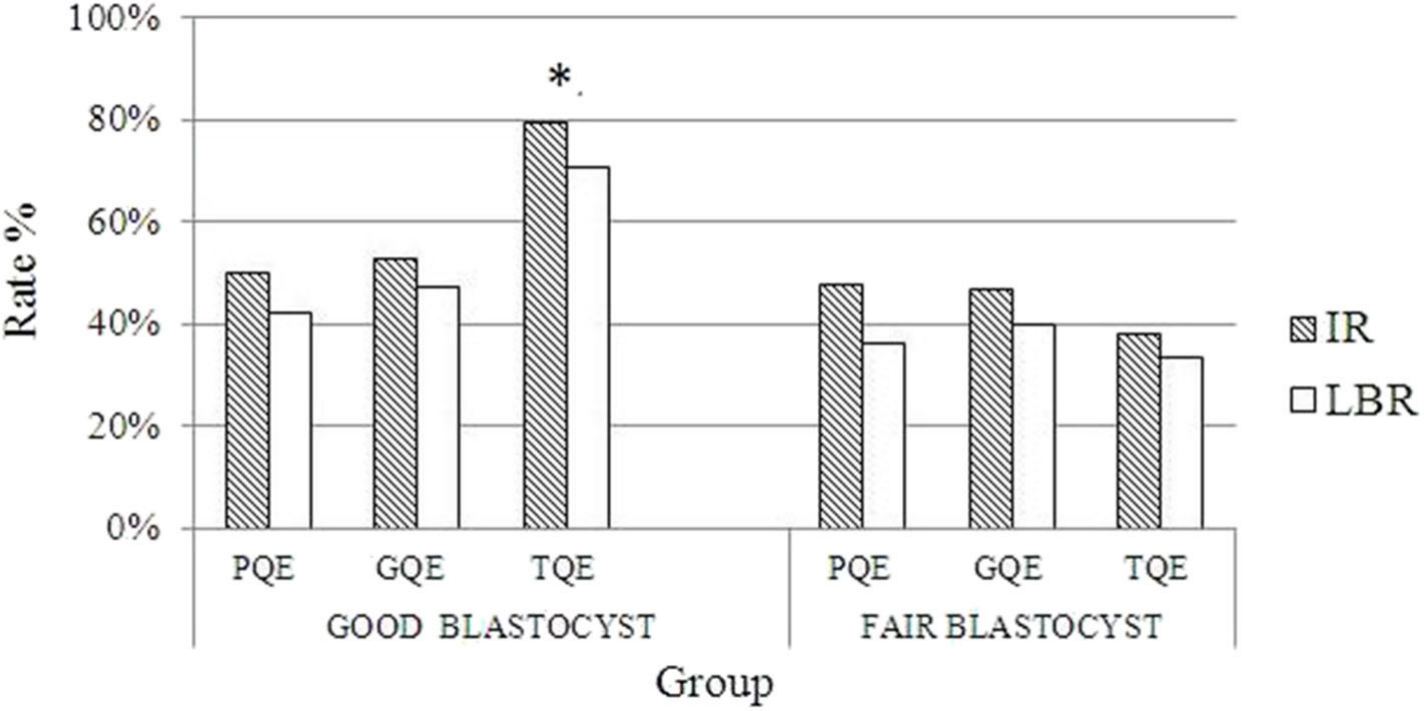
Comparisons of pregnancy outcomes grouped by different qualities of blastocyst and their originated day 3 embryos. *Implantation rate and live birth rate of good blastocysts originated from top quality day 3 embryos is significantly different from that of other groups (*p* < 0.05). PQE, poor quality embryo; GQE, good quality embryo; TQE, top quality embryo; IR, implantation rate; LBR, live birth rate.

Table 4 summarizes the pregnancy outcomes resulting from the transfer of vitrified blastocyst arising from good- and poor-quality cleavage stage embryos. The overall CPR and LBR/transferred embryo were 55.70 and 36.58%, respectively. Finally, no statistically significant difference was found in the CPR, IR and LBR among the three groups. The miscarriage rate was significantly higher in group A (30.16%) than in group C (12.5%, *P* = 0.03). In patients with single embryo transfer, implantation rate and live birth rate were compared based on both blastocysts and its original embryo quality (Figure 1). The IR and LBR of patients transferring single good blastocyst developed from top quality cleavage embryo (79.5 and 70.5%) were significantly higher than other subgroups. There was no statistically differences was displayed in fair blastocyst transferring patients.

**Table 4:** Pregnancy outcomes of the three groups.

	**Group A (*n* = 111)**	**Group B (*n* = 235)**	**Group C (*n* = 119)**	***P*-value**
HCG positive rate	70/111 (63.1%)	152/235 (64.7%)	83/119 (69.8%)	0.40
Clinical pregnancy rate	63/111 (56.8%)	124/235 (52.8%)	72/119 (60.5%)	0.37
Implantation rate	68/148 (46.0%)	137/296 (46.3%)	78/152 (51.3%)	0.55
Twin pregnancy rate	5/63 (7.9%)	11/124 (8.9%)	6/72 (8.3%)	0.98
Triplet pregnancy rate	0/63 (0)	1/124 (0.8%)	0/72 (0)	0.58
Live birth rate/embryo transferred	46/148 (31.1)	105/296 (35.5)	67/152 (44.1)	0.06
Miscarriage rate^*^	19/63 (30.2%)	25/124 (20.2%)	9/72 (12.5%)	0.03
Ectopic pregnancy rate	2/63 (3.2%)	3/124 (2.4%)	1/72 (1.4%)	0.78

* *Miscarriage rate of group A is significantly different from that of group C (p < 0.05)*.

*HCG, human chorionic gonadotrophin*.

### Perinatal Outcomes

Of the 218 live births, no differences in the mean birth weight, gestational age, growth restriction, or PTD were noted among the three groups. No neonatal complications were recorded in the three groups (Table 5).

**Table 5:** Obstetric and perinatal outcomes of the three groups.

	**Group A**	**Group B**	**Group C**	***P*-value**
Gestational age (weeks)	38.2 ± 2.2	38.0 ± 2.3	38.0 ± 2.7	0.68
Preterm delivery rate	6/46 (14.3%)	18/105 (18.8%)	8/67 (12.9%)	0.60
Mean birth weight (g)	3160 ± 617	3208 ± 629	3273 ± 605	0.22
Low birth weight rate	6/46 (13.0%)	17/105 (16.2%)	9/67 (13.4%)	0.83
Congenital anomalies	0	0	0	
Perinatal mortality	0	0	0	

## Discussion

As the expenses of IVF and maximizing the number of viable embryos for transfer are important considerations, extended culture of PQE at cleavage stage may provide infertile couples more opportunities for pregnancies. Although the size of the number of treatment cycles was small, the clinical value is addressed as blastocysts arising from poor-quality cleavage stage embryos can result in comparable LBR, but with an increased miscarriage rate.

### Extended Culture of PQE

Overall, the morphology of cleavage stage embryos had the predictive values for further implantation potential. Until now, the number of cells and fragmentation grade have been considered the most important factors for the embryo scoring system (9). However, the morphological criteria for cleavage embryo selection may be limited as some criteria are still based on the maternal genome. The embryonic genome is fully activated after the 8-cell stage, and the developmental potential is restored during the subsequent cultured process (10). Previously, we discarded poor-quality cleavage stage embryos, based on their supposedly low implantation potential. As this practice results in the loss of viable supernumerary embryos, some clinical studies had suggested that extended cultured PQE could develop into the blastocyst stage and provide good results after warming. Guerif et al. (11) studied young patients poor cleavage stage embryos and found that 78% of the patients achieved a blastocyst transfer and the IR of the blastocysts developed from PQE was 40%. The results of the study by Kaartinen et al. (3) showed 19.7% PQE reached blastocyst stage and were eligible for vitrification after extended culture. According to the reports of Shaw-Jackson et al. (4) and Ren et al. (12), 16 and 6.6%, respectively, of the blastocysts that originated from PQE were vitrified, which may reflect the selection criteria of viable blastocysts. Sallem et al. (13) classified embryo on day 2 and they reported the blastulation of PQE as 48.7%, but this included all blastocyst quality stages. Comparable with results described by Kaartinen et al. (3), extended culture of PQE achieved a viable blastulation rate of 22.5% in our study. Clearly, the extended culturing of PQE could allow for identification of viable blastocysts reducing embryo wastage.

Blastocysts beyond the genomic activation after the 8-cell stages of fertilization are considered preimplantation embryos. Mosaicism and aneuploidies are common in cleavage stage embryos, but this does not necessarily define the embryo development potential. An interesting study from Balaban et al. (14) showed that blastocysts from PQE had a higher IR than similar quality cleavage stage embryos during transfer. This can be explained by the embryo plasticity, the proportion of chromosomally abnormal cells and the corrupted cells that can be eliminated during the extended culture. In line with this view, Fragile et al. (15) reported a lower aneuploidity rate in the blastocyst stage compared to the high rate of complex aneuploidity in the cleavage stage embryos.

### Pregnancy Outcomes of the Blastocysts Originating From Poor-Quality Day 3 Embryos

Recent research shows that pregnancy outcomes are not influenced by determining good or poor-quality at day 2 or day 3 when good-quality blastocysts are transferred at day 5 (6), for both fresh and frozen blastocysts transfer cycles being included. Kaartinen et al. (3) analyzed the outcomes of 134 vitrified-warmed blastocyst transfers that originated from PQE at day 2 or day 3. These transfers resulted in 33 clinical pregnancies (24.6% per ET) and 23 deliveries (17.2% per ET) (3). However, they did not display the outcomes of transferring blastocysts originating from good-quality cleavage stage embryos. Poulain et al. (16) carried out a prospective study including 33 blastocysts transfer cycles. Thirty-four blastocysts originating from PQE yielded ten clinical pregnancies and five live births. In agreement with the previous studies, the present study showed that blastocysts from PQE resulted in similar CPR and IR compared to blastocysts from good or top embryos. In order to investigate which parameter regarding the quality of day 3 embryos or blastocysts was related to pregnant outcome, we compared IR and LBR based on both different blastocysts and day 3 embryos quality. The data displayed blastocysts result in similar IR and LBR, regardless of their quality or origination, except that those good blastocysts originating from top quality embryos on day 3, which resulted in greatly higher IR and LBR. To the best of our knowledge, the present study was the first to compare the implantation potential of frozen-thawed blastocysts based on not only its own score but also its origination. All patients with uterine pathology affecting embryo implantation were excluded. They received the same endometrium preparation protocol, which reduced the heterogeneity of the patients' endometrium receptivity.

In our study, all patients without history of pregnancy loss were included. However, we noticed a significantly higher incidence of miscarriages (30.16%) in group A than the top embryo group. There were conflicting conclusions regarding the miscarriage rate of transferring PQE. Our results are comparable with those of Poulain (16) who reported a CPR of 30.3% and spontaneous abortion rate of 50% for poor-quality cleavage stage embryos with extended culture. It is possible that differences in miscarriage rates may be attributed to a high prevalence of chromosomal abnormalities in blastocyst originating from the PQE. However, Kirillova et al. (17) found a lower miscarriage rate in transfer cycles with PQE compared to the programs with good and fair quality embryos. The authors speculated that aneuploid embryos of good and fair quality are able to implant due to the superior trophectoderm morphology.

As chromosomal abnormality is one of the main reasons for early spontaneous abortions, the explanation for the elevated miscarriage rate in Group A could be the higher incidence of chromosomal abnormalities in the blastocyst population derived from poor quality embryos. Also, a diagnosis of complex aneuploidy was associated with blastocysts morphology (18). In 2017, Majumdar et al. (19) reported the euploidy rate was 73.2, 50, and 40.5% in the good, average and poor morphology blastocyst groups, respectively (*P* = 0.001). However, no significant association was found between the morphologies of day 3 embryos and euploidy rates. Compared to good-quality cleavage stage embryos, PQE are often associated with poor blastocyst morphologies. We also found a lower good-quality blastulation rate in the PQE group compared to the top embryo group (4.8 vs. 8.4%). Although the scores of transferred blastocysts were not different among the three groups, we suggest developing additional morphokinetic markers for selecting good-quality blastocysts to reduce the miscarriage rate.

In addition, continuous monitoring via time-lapse imaging is recommended to evaluate an embryos potential for implantation without the need to remove the embryo from optimal culture conditions. Chromosomally normal and abnormal embryos may display different kinetic activities (20). Euploid embryos required a significantly shorter time to reach initiation of compaction, time to start blastulation, and to full blastulation (21). Motato et al. (22) recently described a hierarchical classification system of blastocysts according to the following morphokinetic parameters: (i) the time of morulation (tM), and (ii) timing of transition from 5-blastomere embryo to until eight-blastomere embryo (t8–t5). The combination of extended culturing of PQE and a time-lapse imaging technique should be further evaluated in a prospective way.

### Perinatal Outcomes of Blastocysts Arising From Day 3 PQE

Oron et al. (23) reported in fresh ET cycles, for cleavage embryo or blastocysts, no increased risk of maternal or neonatal complications were observed with the transfer of PQE. In agreement with their findings, our results showed that when pregnancy is achieved, there is a similar probability in perinatal outcomes following the transfer of frozen-thawed blastocysts arising from PQE and there was no difference in the PTD, LBW, and congenital malformations among the three groups. In a meta-analysis, among IVF/ICSI singleton pregnancies, pooled estimates of total congenital malformations were 4.84% (24). The present study has a limitation of the small sample size, not enough to clarify the effect of transferring blastocysts arising from PQE on birth defects. A recent single-center retrospective cohort study displayed that the higher ICM quality of blastocysts among FET cycles was associated with increased chance of preterm birth (25). Therefore, further research is required to ensure the safety of transfer of blastocysts arising from PQE.

### Limitations

There are limitations to this study. It is a retrospective unicenter study and the number of embryos included may affect the statistical power. Moreover, age is an important element in evaluating pregnancy outcomes of IVF and stratified analysis based on the age of female patients may result in more reliable conclusions. In addition, HRT cycle are associated with a higher rate of miscarriage in general in FET (26, 27), so we need more information concerning the endometrial preparation protocols to confirm the conclusions.

## Conclusion

The results of this small-sample study showed that blastocysts arising from PQE resulted in the similar CPR and LBR but higher miscarriage rate compared with those from good- and top-quality embryos. In addition, blastocysts arising from PQE were not associated with increased adverse perinatal outcomes. As a summary, extended culture of poor-quality cleavage stage embryos provides a clinical strategy for avoiding embryo wastage and reducing costs. Future research to identify non-invasive biomarkers of reproductive potential may further enhance blastocysts selection.

## Data Availability Statement

The raw data supporting the conclusions of this article will be made available by the authors, without undue reservation.

## Ethics Statement

The treatment was conducted after informed consent was obtained from the patients and was carried out according to the guidelines of the Ministry of Public Health of China. It was approved by the reproductive ethics committee at the Medical School of Shanghai Jiaotong University.

## Author Contributions

LX conducted the analysis and wrote the manuscript. SZ and HX collected the data. XW statistically analyzed the data. ZN supervised the study concept and reviewed the manuscript. AZ reviewed the manuscript. All authors contributed to the article and approved the submitted version.

## Conflict of Interest

The authors declare that the research was conducted in the absence of any commercial or financial relationships that could be construed as a potential conflict of interest.
