# Corrigendum: Miscarriage Rate Is High With Frozen-Thawed Blastocysts Arising From Poor-Quality Cleavage Stage Embryos

**DOI:** 10.3389/fendo.2020.613921

**Published:** 2020-11-26

**Authors:** Lan Xia, Shen Zhao, Huiui Xu, Xian Wu, Aijun Zhang, Zhihong Niu

**Affiliations:** Reproductive Medical Center of Ruijin Hospital, School of Medicine, Shanghai Jiao Tong University, Shanghai, China

**Keywords:** blastocysts, embryo, miscarriage rate, perinatal outcomes, clinical pregnancy rate

Co-author Aijun Zhang was not included as a co-corresponding author in the published article.

The authors apologize for this error and state that this does not change the scientific conclusions of the article in any way. The original article has been updated.

